# MCL1 Promotes Endothelial Senescence and Atherosclerosis via Glycolytic Reprogramming-Induced H4K12 Lactylation

**DOI:** 10.3390/ijms27167392

**Published:** 2026-08-18

**Authors:** Yang Li, Ling Li, Hongxia Gao, Yakun Gao, Wanying Wu, Longhua Fan

**Affiliations:** Institute of Vascular Surgery, Zhongshan Clinical Medical College, Fudan University, Shanghai 200032, China; 23111210156@m.fudan.edu.cn (Y.L.); 17210700117@fudan.edu.cn (L.L.); 23111350002@m.fudan.edu.cn (H.G.); 24111210169@m.fudan.edu.cn (Y.G.); 25111210202@m.fudan.edu.cn (W.W.)

**Keywords:** MCL1, endothelial senescence, atherosclerosis, glycolysis, histone lactylation

## Abstract

Accumulation of senescent endothelial cells (ECs) accelerates atherosclerosis, yet the molecular orchestrators linking metabolic dysregulation to epigenetic reprogramming during EC senescence remain elusive. Here, we investigated the specific role of Myeloid cell leukemia 1 (MCL1) in endothelial senescence and atherogenesis. Through integrative bioinformatics, MCL1 was identified as a candidate senescence regulator. MCL1 expression was validated in human carotid atherosclerotic plaques, ApoE^−/−^ mice, and multiple EC senescence models, primarily HRAS^G12V^-induced senescent human umbilical vein endothelial cells (HUVECs). Loss- and gain-of-function assays were performed in HUVECs. Underlying metabolic and epigenetic mechanisms were explored using Seahorse extracellular flux analysis, lactate measurements, Co-IP and CUT&Tag sequencing. In vivo therapeutic potential was evaluated via AAV-mediated MCL1 knockdown in high-fat diet-fed ApoE^−/−^ mice. MCL1 expression was significantly upregulated in atherosclerotic plaques and senescent ECs. Functionally, MCL1 knockdown attenuated senescence markers (SA-β-gal, P21), suppressed the senescence-associated secretory phenotype (SASP), and restored cell proliferation, whereas MCL1 overexpression exacerbated EC senescence. Mechanistically, MCL1 promoted glycolytic reprogramming and intracellular lactate accumulation. This metabolic byproduct served as an epigenetic substrate for histone H4K12 lactylation (H4K12la), which specifically enriched at the *CDKN1A* (P21) promoter, correlating with its transcription. In vivo, AAV-mediated MCL1 silencing effectively reduced vascular H4K12la levels, alleviated vascular senescence, and substantially constrained atherosclerotic lesion areas. Our findings identify MCL1 as a pivotal regulator of endothelial senescence and atherosclerosis, operating through a metabolic-\–epigenetic mechanism involving glycolytic reprogramming, lactate accumulation, and H4K12la-associated P21 upregulation. These findings suggest that targeting the MCL1-associated pathway may represent a potential therapeutic strategy for atherosclerosis.

## 1. Introduction

Atherosclerosis is a chronic inflammatory disease of the arterial wall and represents the principal pathological basis for the majority of cardiovascular events [1], including myocardial infarction [2], ischemic stroke [3], and peripheral artery disease [4]. Despite substantial advances in lipid-lowering therapies, antiplatelet agents, and interventional techniques, atherosclerotic cardiovascular disease remains the leading cause of morbidity and mortality worldwide [1]. This persistent clinical burden highlights the urgent need to elucidate novel molecular mechanisms underlying atherogenesis and to identify new therapeutic targets.

The vascular endothelium forms a dynamic interface between circulating blood and the arterial wall and is central to vascular homeostasis [5], It regulates vasomotor tone, permeability, coagulation, and inflammation with rapid, context-dependent responses [6]. Endothelial dysfunction is widely recognized as the earliest event in atherogenesis [7]. This initial damage triggers a cascade of pathological processes, including lipid retention, monocyte infiltration, and neointimal formation [8,9,10]. Cellular senescence drives much of this endothelial dysfunction. It accelerates vascular aging and atherosclerosis [11]. Senescent endothelial cells build up inside atherosclerotic plaques. They display several clear hallmarks [12], including irreversible cell cycle arrest, upregulated expression of cyclin-dependent kinase inhibitors (P21 and P16), increased senescence-associated β-galactosidase (SA-β-gal) activity [13], and a pro-inflammatory secretory profile known as the senescence-associated secretory phenotype (SASP) [14]. These cells worsen local inflammation, damage vascular repair, and push plaques toward progression and instability [12,15]. Clearing senescent cells or blocking the senescent phenotype has reduced atherosclerosis in preclinical models [13,16]. Such results point to real therapeutic potential in targeting endothelial senescence. Yet in practice, the upstream molecular regulators responsible for initiating and sustaining endothelial senescence in the atherosclerotic microenvironment remain incompletely understood, leaving clinicians and investigators without clear molecular entry points for timely, endothelium-directed intervention in human-like disease settings.

Myeloid cell leukemia-1 (MCL1) is an anti-apoptotic member of the BCL-2 protein family. It sits anchored in the outer mitochondrial membrane, where it blocks mitochondrial outer membrane permeabilization and prevents cytochrome c release. MCL1 is essential for embryonic development [17]. It also keeps many cell types alive, including cardiomyocytes [18], hematopoietic stem cells [19], and lymphocytes [20]. In cancer, MCL1 is frequently overexpressed and this overexpression drives tumor progression and chemoresistance [21,22], which is why MCL1 has become a major therapeutic target in oncology. Beyond its canonical anti-apoptotic function [23], accumulating evidence has revealed non-canonical roles for MCL1 in regulating mitochondrial morphology, cristae organization, oxidative phosphorylation, and cellular bioenergetics [24,25,26]. Emerging studies indicate that MCL1 also modulates cytosolic metabolism. For instance, recent work in oncology has demonstrated that MCL1 can directly interact with hexokinase 2 (HK2) to promote glycolysis, thereby supporting the survival of acute myeloid leukemia cells [27]. Metabolic reprogramming occurs in both tumors and senescent cells; we hypothesized that aberrant accumulation of MCL1 in stressed endothelial cells may exert similar non-canonical metabolic functions. Whether MCL1 drives endothelial senescence by reprogramming glycolysis during atherosclerosis progression, however, remains largely unexplored.

Metabolic reprogramming is a well-recognized hallmark of cellular senescence [28]. Senescent cells usually show mitochondrial dysfunction. They cut back oxidative phosphorylation and shift toward higher glycolysis. This change drives elevated lactate production. Once considered merely a metabolic byproduct, lactate is now recognized as a bioactive signaling molecule that takes part in diverse physiological and pathological processes. A landmark discovery by Zhang and colleagues in 2019 identified histone lysine lactylation (Kla) as a novel epigenetic modification. This mark directly ties cellular metabolism to gene expression [29]. Subsequent studies have implicated histone lactylation marks—including H3K18la and H4K12la—in macrophage polarization, tumor progression, embryonic development, and immune regulation [30,31,32]. Emerging evidence has also begun to associate histone lactylation with cardiovascular pathologies such as cardiac remodeling, ischemic injury, and vascular inflammation [33]. Nevertheless, whether histone lactylation contributes to endothelial senescence and atherosclerosis, and whether specific lactylation sites can be therapeutically targeted in vascular disease, remain important unresolved questions.

In the present study, through integrated bioinformatics analysis of transcriptomic, senescence-associated, and mitochondrial gene databases, we identified MCL1 as a previously unrecognized regulator of endothelial senescence in atherosclerosis. We confirmed MCL1 upregulation in human atherosclerotic plaques, in the aortas of ApoE^−/−^ mice, and in senescence-induced human umbilical vein endothelial cells (HUVECs). Using complementary loss- and gain-of-function approaches in vitro, together with AAV-mediated endothelial-specific gene silencing in vivo, we systematically evaluated the functional role of MCL1 in endothelial senescence and atherogenesis. Mechanistically, we demonstrate that MCL1 drives a metabolic–epigenetic cascade involving enhanced glycolysis, lactate accumulation, and H4K12la-mediated transcriptional activation of P21. Our findings reveal a previously unappreciated MCL1-driven metabolic–epigenetic axis that governs endothelial senescence and atherosclerosis, suggesting that targeting this axis may represent a promising therapeutic strategy for atherosclerotic vascular disease.

## 2. Results

### 2.1. MCL1 Expression Is Significantly Upregulated in Atherosclerotic Plaques and Senescent Endothelial Cells

**Figure 1:**
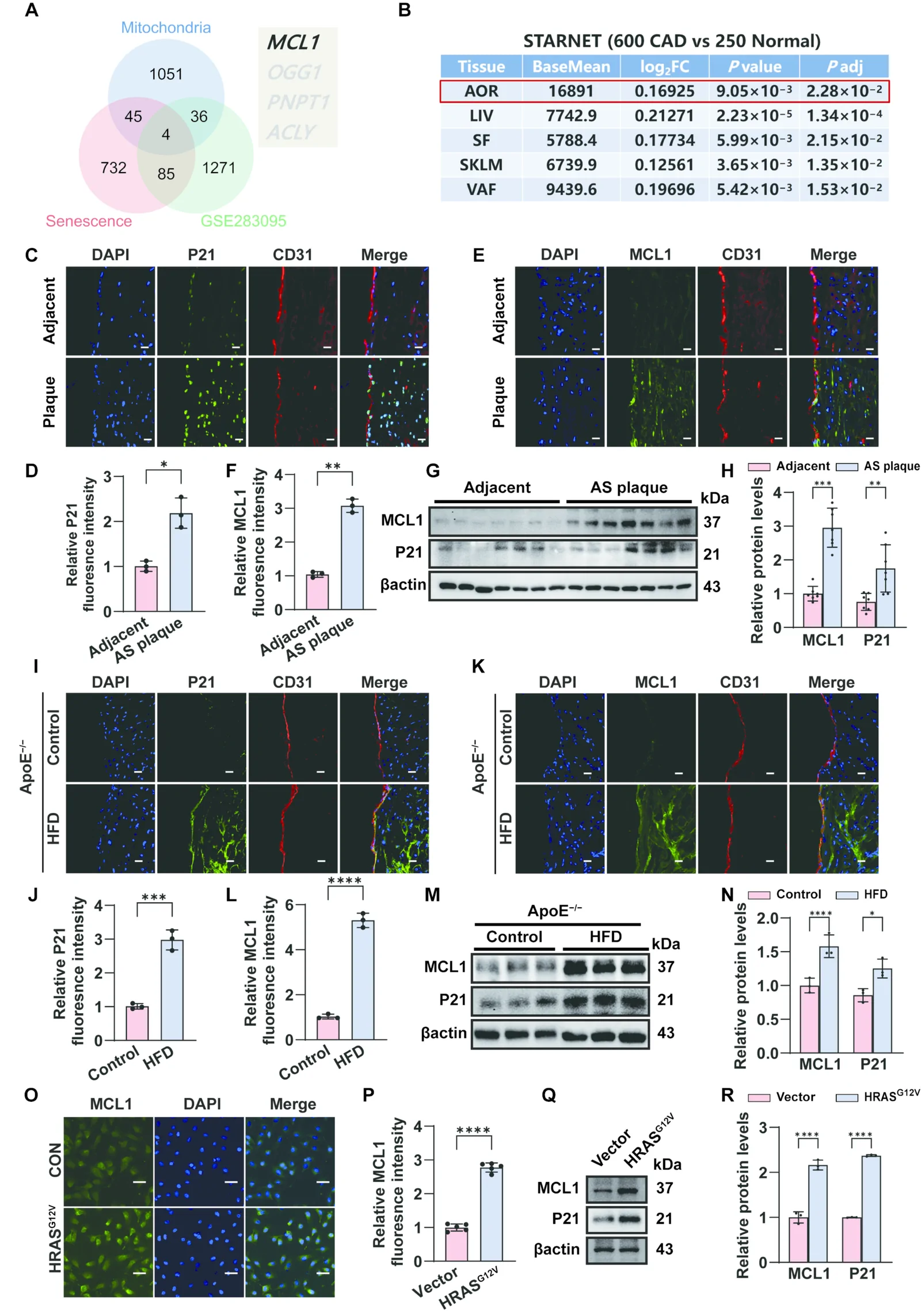
MCL1 is upregulated in atherosclerotic plaques and in senescent endothelial cells. (**A**) Venn diagram showing the intersection of GSE283095, CellAge database, and MitoMiner Database, identifying *MCL1* as a candidate gene. (*MCL1*: Myeloid cell leukemia-1; *OGG1*: 8-Oxoguanine DNA Glycosylase; *PNPT1*: Protein tyrosine phosphatase non-receptor type 1; *ACLY*: ATP Citrate Lyase). (**B**) MCL1 mRNA expression is significantly elevated in atherosclerotic aortic tissues from coronary artery disease patients compared with non-atherosclerotic controls in the STARNET database. AOR: aortic root; LIV: liver; SF: subcutaneous fat; SKLM: skeletal muscle; VAF: visceral abdominal fat. (**C**,**D**) Representative immunofluorescence images and corresponding quantification analysis within CD31^+^ endothelial regions showing the increased accumulation of senescent endothelial cells in the core region of human carotid atherosclerotic plaques. (*n* = 3) (DAPI, blue; p21, green; CD31, red). Scale bar 200 μm. (P21, Adjacent vs. AS plaque, *p* = 0.0232). (**E**,**F**) Representative immunofluorescence images and quantitative analysis within CD31^+^ endothelial regions revealing the elevated expression of MCL1 in the core region of human carotid atherosclerotic plaques. (*n* = 3) (DAPI, blue; MCL1, green; CD31, red). Scale bar 200 μm. (MCL1, Adjacent vs. AS plaque, *p* = 0.0056). (**G**,**H**) Representative Western blot images and quantification of MCL1 and P21 protein expression levels in the core regions of human carotid atherosclerotic plaques. (MCL1, Adjacent vs. AS plaque, *p* = 0.0002; P21, Adjacent vs. AS plaque, *p* = 0.0094). (**I**,**J**) Representative immunofluorescence staining and quantitative analysis of the CD31-positive regions showing the increased accumulation of senescent endothelial cells in the atherosclerotic vascular tissues of ApoE^−/−^ mice. (*n* = 3 independent biological replicates) (DAPI, blue; P21, green; CD31, red). Scale bar 200 μm. (P21, Control vs. HFD, *p* = 0.0004). (**K**,**L**) Representative immunofluorescence images and quantitative analysis of the CD31-positive regions revealing the elevated expression of MCL1 in the atherosclerotic vascular tissues of ApoE^−/−^ mice. (*n* = 3 independent biological replicates) (DAPI, blue; MCL1, green; CD31, red). Scale bar 200 μm. (MCL1, Control vs. HFD, *p* < 0.0001). (**M**,**N**) ApoE^−/−^ mice aortas: representative immunoblots (**M**) and densitometric quantification (**N**) demonstrating increased MCL1 and P21 protein levels in atherosclerotic tissue. (MCL1, Control vs. HFD, *p* < 0.0001; P21, Control vs. HFD, *p* = 0.0132). (**O**–**R**) HRAS^G12V^-induced senescence in HUVECs increases MCL1. All bars represent mean ± SD.* *p* < 0.05; ** *p* < 0.01; *** *p* < 0.001; **** *p* < 0.0001. (**D**,**F**,**H**) were analyzed by paired *t*-test, (**J**,**L**,**N**,**R**) were analyzed by unpaired *t*-test.

To identify candidate genes implicated in endothelial cell senescence during atherosclerosis, we performed an integrative bioinformatic analysis by intersecting differentially expressed genes from the atherosclerosis-related GSE283095 dataset with senescence-associated genes from the CellAge database (https://genomics.senescence.info/cells/ (accessed on 10 December 2024)) and mitochondrial proteins curated in the MitoMiner database (http://mitominer.mrc-mbu.cam.ac.uk/ (accessed on 10 December 2024)). The Venn diagram identified MCL1 as a candidate gene linking mitochondria, senescence, and atherosclerosis (Figure 1A). Further interrogation of the STARNET (Stockholm–Tartu Atherosclerosis Reverse Network Engineering Task) database (http://starnet.mssm.edu/ (accessed on 10 December 2024)) confirmed that MCL1 mRNA expression was significantly elevated in the atherosclerotic aortas of patients with coronary artery disease relative to healthy controls (Figure 1B).

To validate these clinical findings, we collected human carotid atherosclerotic plaques. Immunofluorescence and Western blot analyses demonstrated a marked accumulation of senescent endothelial cells (ECs) within the core regions of human plaques, as evidenced by increased P21 expression specifically co-localized with the endothelial marker CD31 (Figure 1C,D). Concomitantly, MCL1 protein expression was robustly upregulated within the same plaque regions (Figure 1E–H). These clinical observations were faithfully recapitulated in vivo in ApoE^−/−^ mice, in which atherosclerotic vessels exhibited parallel increases in endothelial senescence and MCL1 expression (Figure 1I–N). Furthermore, in vitro experiments employing HRAS^G12V^-induced premature senescence in HUVECs confirmed that MCL1 expression was intrinsically elevated upon senescence induction (Figure 1O–R). Collectively, these data indicate that MCL1 is highly expressed in senescent ECs and atherosclerotic lesions.

### 2.2. MCL1 Acts as a Pivotal Regulator of Endothelial Cell Senescence

**Figure 2:**
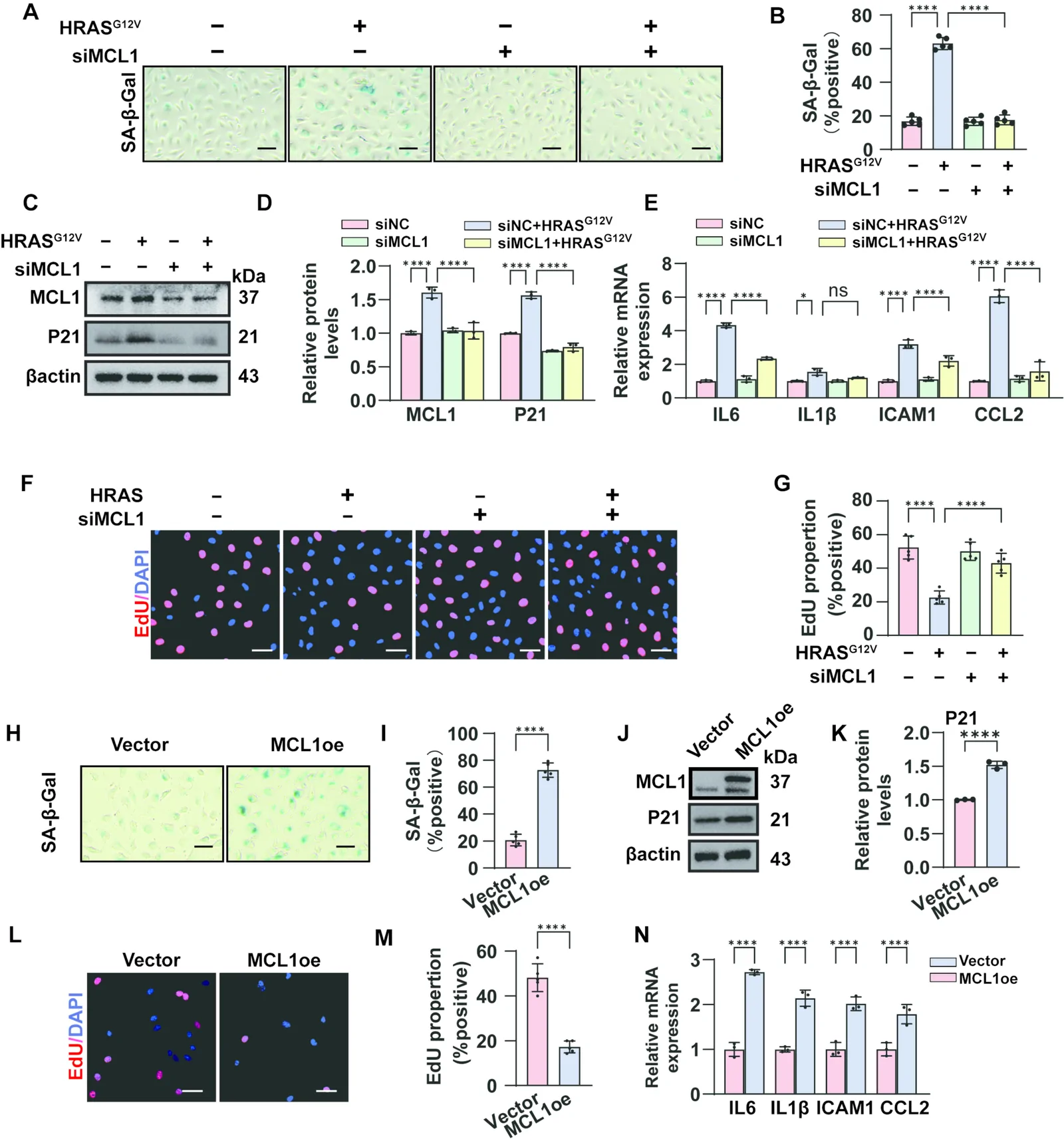
MCL1 regulates senescence in human endothelial cells. (**A**,**B**) SA-β-gal staining of senescence-induced HUVECs transfected with control siRNA or siMCL1: representative images (**A**) and quantification of SA-β-gal-positive cells (**B**) showing that MCL1 knockdown reduces cellular senescence (siNC vs. siNC + HRAS^G12V^, *p* < 0.0001, siNC + HRAS^G12V^ vs. siMCL1 + HRAS^G12V^, *p* < 0.0001). (*n* = 5 independent biological replicates). Scale bar 100 μm. (**C**,**D**) Immunoblot (**C**) and densitometric quantification (**D**) showing decreased p21 protein levels in senescence-induced HUVECs following MCL1 knockdown. (**E**) qRT-PCR showing reduced SASP mRNA levels in senescence-induced HUVECs after MCL1 knockdown. (IL1β, siNC vs. siNC + HRAS^G12V^, *p* = 0.0411, siNC + HRAS^G12V^ vs. siMCL1 + HRAS^G12V^, *p* = 0.9963; IL6, ICAM1, CCL2, siNC vs. siNC + HRAS^G12V^, *p* < 0.0001, siNC + HRAS^G12V^ vs. siMCL1 + HRAS^G12V^, *p* < 0.0001). (**F**,**G**) EdU incorporation assay in senescence-induced HUVECs with siMCL1: representative images (**F**) and quantification of EdU-positive cells (**G**) demonstrating an increased fraction of proliferating cells. (siNC vs. siNC + HRAS^G12V^, *p* < 0.0001, siNC + HRAS^G12V^ vs. siMCL1 + HRAS^G12V^, *p* < 0.0001) (*n* = 5 independent biological replicates) (DAPI, blue; EdU, red). Scale bar 50 μm. (**H**,**I**) SA-β-gal staining in HUVECs overexpressing MCL1: representative images (**H**) and quantification (**I**) showing increased senescence. (*n* = 5 independent biological replicates). Scale bar 100 μm. (**J**,**K**) Immunoblot (**J**) and quantification (**K**) showing elevated p21 protein upon MCL1 overexpression. (**L**,**M**) EdU assay in HUVECs overexpressing MCL1: representative images (**L**) and quantification (**M**) showing a reduced fraction of EdU-positive cells. (*n* = 5 independent biological replicates) (DAPI, blue; EdU, red). Scale bar 50 μm. (**N**) qRT-PCR showing increased SASP mRNA levels with MCL1 overexpression. All bars represent mean ± SD. ns, no significance; * *p* < 0.05; **** *p* < 0.0001. (**B**,**D**,**E**,**G**) were analyzed by two-way ANOVA followed by Tukey’s post hoc test. (**I**,**K**,**M**,**N**) were analyzed by unpaired *t*-test.

Given the strong correlation between MCL1 and endothelial senescence, we next investigated its functional role in vitro. Knockdown of MCL1 with a specific siRNA (siMCL1) in senescence-induced HUVECs significantly reversed the senescence phenotype, as evidenced by a reduced proportion of SA-β-gal-positive cells (Figure 2A,B), decreased P21 protein levels (Figure 2C,D), and downregulated mRNA expression of senescence-associated secretory phenotype (SASP) factors (Figure 2E). In addition, EdU incorporation assays revealed that MCL1 knockdown restored endothelial proliferative capacity (Figure 2F,G). To further determine whether MCL1 upregulation is also involved in non-oncogenic senescence, we established an oxidative stress-induced senescence model using tert-butyl hydroperoxide (TBHP). TBHP treatment induced a typical senescent phenotype in HUVECs, while MCL1 knockdown markedly attenuated senescent phenotype in HUVECs (Figure S1A–G). In addition, oxidized low-density lipoprotein (oxLDL) treatment increased MCL1 protein expression in HUVECs (Figure S1I), although robust senescence phenotypes were not observed under the current experimental conditions.

Conversely, overexpression of MCL1 was sufficient to induce senescence in normal HUVECs, as reflected by increased SA-β-gal activity, upregulated P21 and SASP levels, and impaired proliferation (Figure 2H–N). Collectively, these gain- and loss-of-function studies establish MCL1 as a critical driver of endothelial senescence.

### 2.3. AAV-Mediated MCL1 Knockdown Attenuates Atherosclerosis and Reduces Endothelial Senescence In Vivo

**Figure 3:**
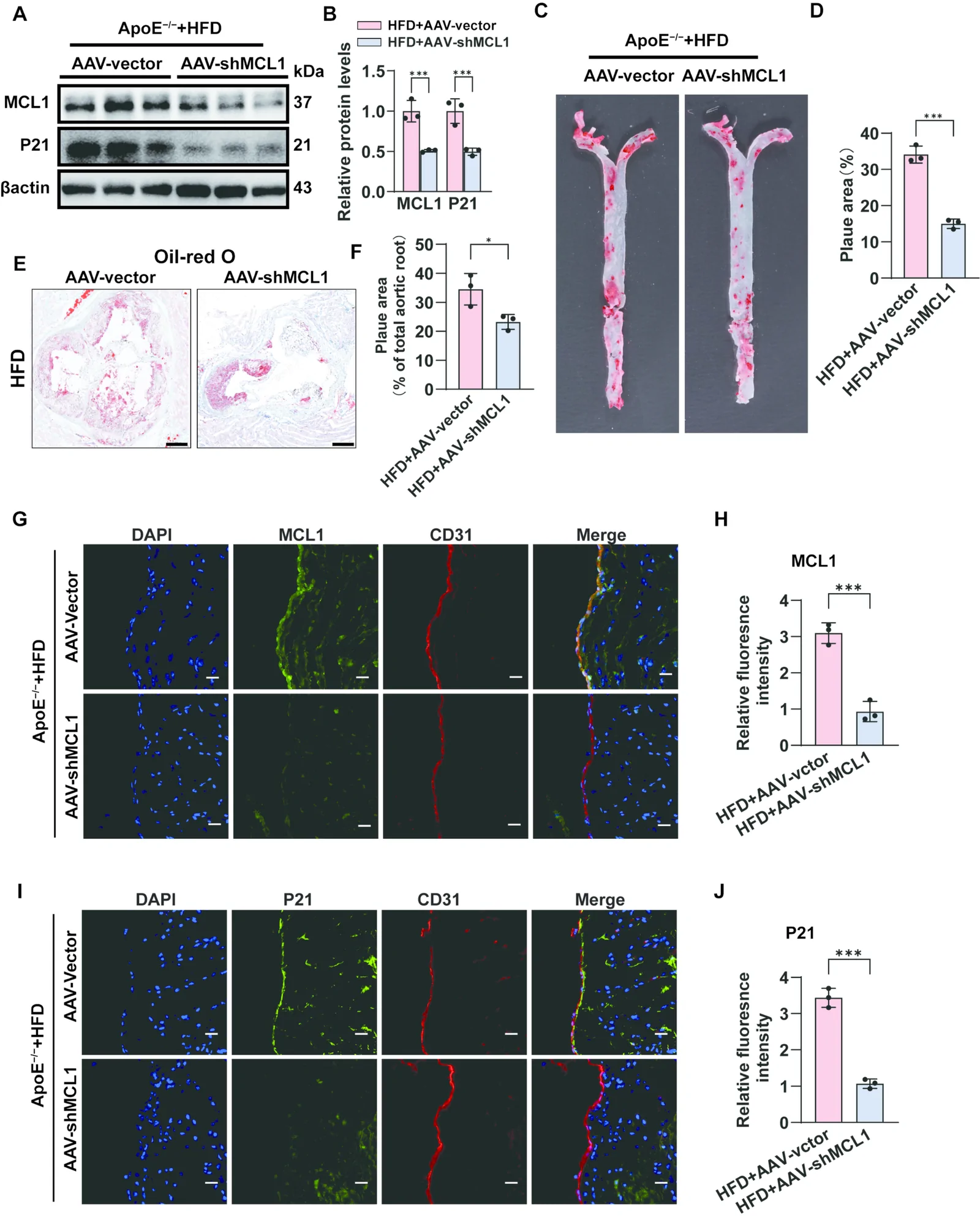
AAV-mediated MCL1 knockdown alleviates atherosclerosis and reduces endothelial senescence in ApoE^−/−^ mice. (**A**,**B**) Western blot analysis and quantification showing that AAV-mediated MCL1 knockdown significantly reduces P21 protein expression in aortic tissues of atherosclerotic ApoE^−/−^ mice. (MCL1, AAV-vector vs. AAV-shMCL1, *p* = 0.0032; P21, AAV-vector vs. AAV-shMCL1, *p* = 0.0051) (*n* = 3 independent biological replicates). (**C**–**F**) Oil Red O staining of en face aortas (**C**) and aortic root cross-sections (**E**) demonstrating that MCL1 knockdown attenuates atherosclerotic plaque burden in ApoE^−/−^ mice. Quantitative analyses of lesion areas are shown in (**D**) (AAV-vector vs. AAV-shMCL1, *p* = 0.0002) and (**F**) (AAV-vector vs. AAV-shMCL1, *p* = 0.0316) respectively. (*n* = 3 independent biological replicates). (**G**,**H**) Representative immunofluorescence staining and quantification within CD31^+^ endothelial regions showing that AAV-mediated MCL1 knockdown decreases MCL1 expression in the endothelium of atherosclerotic lesions. (AAV-vector vs. AAV-shMCL1, *p* = 0.0007) (*n* = 3 independent biological replicates) (DAPI, blue; MCL1, green; CD31, red). Scale bar, 50 μm. (**I**,**J**) Representative immunofluorescence staining and quantification within CD31^+^ endothelial regions showing that MCL1 knockdown reduces the number of senescent endothelial cells (P21-positive) in atherosclerotic aortas of ApoE^−/−^ mice. (AAV-vector vs. AAV-shMCL1, *p* = 0.0002) (*n* = 3 independent biological replicates) (DAPI, blue; P21, green; CD31, red). Scale bar, 50 μm. All bars represent mean ± SD. * *p* < 0.05; *** *p* < 0.001. Data were analyzed by unpaired *t*-test.

To evaluate the therapeutic potential of targeting MCL1 in atherosclerosis, we employed AAV-mediated knockdown of MCL1 in ApoE^−/−^ mice maintained on a high-fat diet. Western blot analysis confirmed that AAV-shMCL1 effectively reduced P21 protein expression in the vascular tissues of atherosclerotic ApoE^−/−^ mice (Figure 3A,B).

En face Oil Red O staining of whole aortas and Oil Red O staining of aortic root cross-sections revealed that AAV-mediated MCL1 knockdown significantly reduced atherosclerotic lesion burden compared with control mice (Figure 3C–F). Immunofluorescence analysis further demonstrated that AAV-shMCL1 effectively diminished endothelial MCL1 expression in atherosclerotic vessels (Figure 3G,H) and substantially reduced the number of senescent endothelial cells (CD31^+^P21^+^) within the aorta (Figure 3I,J). These results indicate that in vivo silencing of MCL1 attenuates atherosclerotic progression, at least in part by suppressing endothelial cell senescence.

### 2.4. MCL1 Regulates Metabolic Reprogramming in Senescent HUVECs

Given that MCL1 was identified through integration with a mitochondria-related database, we hypothesized that MCL1 might regulate endothelial senescence via metabolic reprogramming. To test this hypothesis, we performed Seahorse extracellular flux analysis to assess mitochondrial respiration and glycolysis.

**Figure 4:**
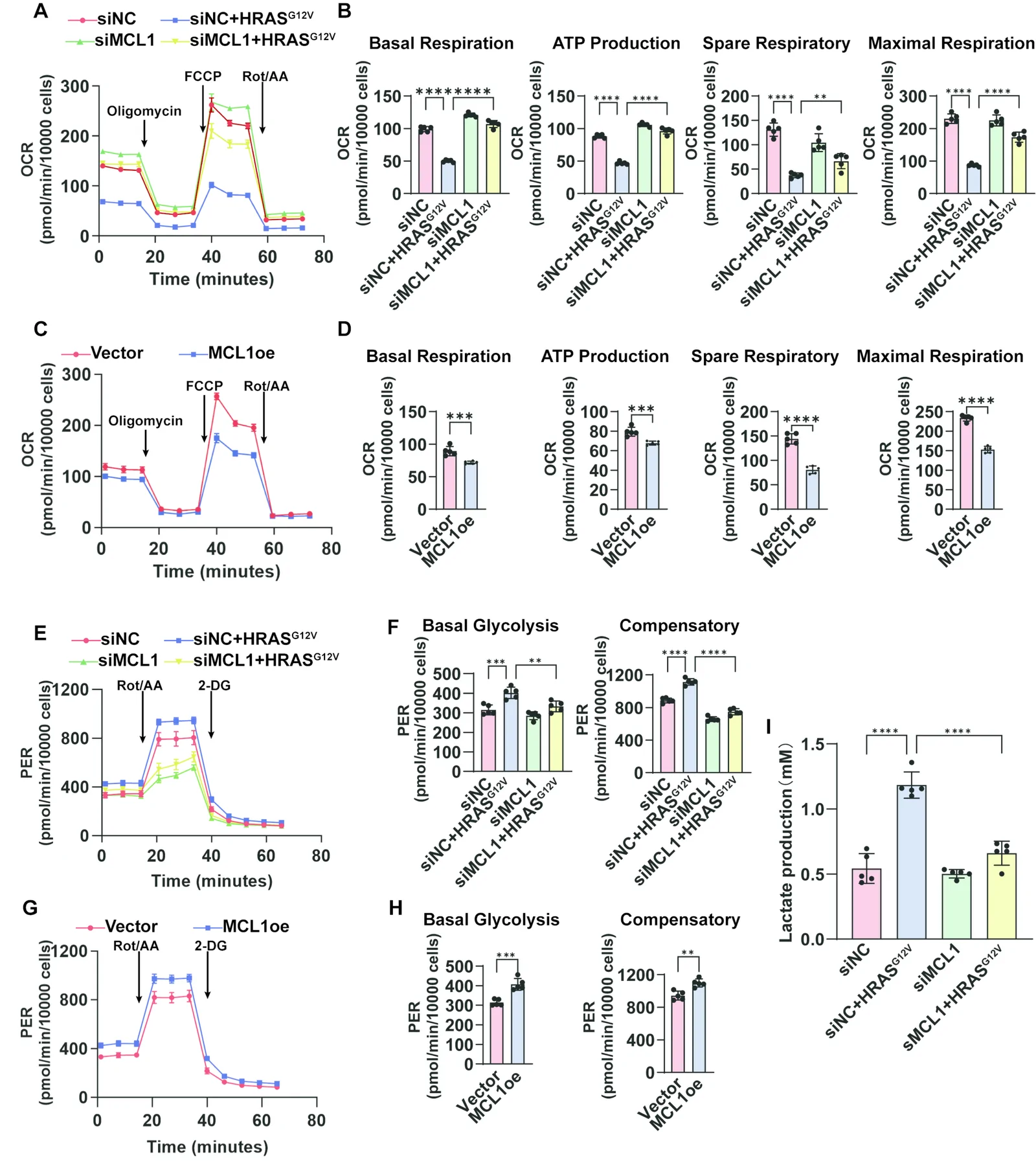
MCL1 regulates metabolic reprogramming in senescent HUVECs. (**A**,**B**) Seahorse extracellular flux analysis showing the oxygen consumption rate (OCR) curves (**A**) and quantification of basal respiration, ATP production, spare respiratory capacity, and maximal respiration (**B**). HRAS^G12V^-induced senescent HUVECs exhibited decreased OCR and reduced basal respiration, ATP production, spare respiratory capacity, and maximal respiration compared with control cells, whereas MCL1 knockdown restored these mitochondrial respiratory parameters in senescent HUVECs. (*n* = 5 independent biological replicates). (**C**,**D**) OCR curves (**C**) and quantification of basal respiration, ATP production, spare respiratory capacity, and maximal respiration (**D**) demonstrating that MCL1 overexpression suppressed mitochondrial respiration in HUVECs. (*n* = 5 independent biological replicates). (**E**,**F**) Seahorse glycolytic rate assay showing the proton efflux rate (PER) curves (**E**) and quantification of basal glycolysis and compensatory glycolysis (**F**). Senescent HUVECs displayed elevated PER, basal glycolysis, and compensatory glycolysis, which were attenuated by MCL1 knockdown. (*n* = 5 independent biological replicates). (**G**,**H**) PER curves (**G**) and quantification of basal glycolysis and compensatory glycolysis (**H**) showing that MCL1 overexpression enhanced glycolytic activity in HUVECs. (*n* = 5 independent biological replicates). (**I**) Measurement of lactate production showing that MCL1 knockdown reduced lactate generation in senescent HUVECs. (*n* = 5 independent biological replicates). All bars represent mean ± SD. ** *p* < 0.01; *** *p* < 0.001; **** *p* < 0.0001. (**B**,**F**,**I**) were analyzed by two-way ANOVA followed by Tukey’s post hoc test. (**D**,**H**) were analyzed by unpaired *t*-test.

Senescent HUVECs exhibited a significantly reduced oxygen consumption rate (OCR), encompassing decreased basal respiration, ATP production, spare respiratory capacity, and maximal respiration, compared with control cells. Notably, MCL1 knockdown restored mitochondrial respiratory function in senescent HUVECs (Figure 4A,B). Conversely, MCL1 overexpression suppressed OCR and diminished all measured mitochondrial respiratory parameters in HUVECs (Figure 4C,D).

We next examined glycolytic activity by measuring the proton efflux rate (PER). Senescence induction markedly elevated PER, basal glycolysis, and compensatory glycolysis in HUVECs, whereas MCL1 knockdown reversed these glycolytic alterations (Figure 4E,F). Consistent with these observations, MCL1 overexpression enhanced glycolytic activity in HUVECs (Figure 4G,H). Furthermore, MCL1 knockdown significantly reduced lactate production in senescence-induced HUVECs (Figure 4I and Figure S1H).

To further explore the mechanism linking MCL1 to glycolytic regulation, we examined several key glycolytic enzymes. MCL1 knockdown selectively reduced PFKP protein expression in senescent HUVECs, whereas the expression levels of HK2, HK1, PKM2, PKM1, PDH, and LDHA were not significantly altered (Figure S3A,B). Co-immunoprecipitation assays further suggested a potential interaction between MCL1 and PFKP in HUVECs (Figure S3C). In addition, immunofluorescence staining demonstrated co-localization of MCL1 and PFKP in HUVECs (Figure S3D). These findings suggest that MCL1 may promote glycolytic remodeling at least partially through regulation of PFKP.

Collectively, these findings demonstrate that MCL1 drives a metabolic switch from oxidative phosphorylation to glycolysis in senescent endothelial cells, accompanied by increased lactate generation.

### 2.5. MCL1 Promotes HUVEC Senescence via Lactate Accumulation

**Figure 5:**
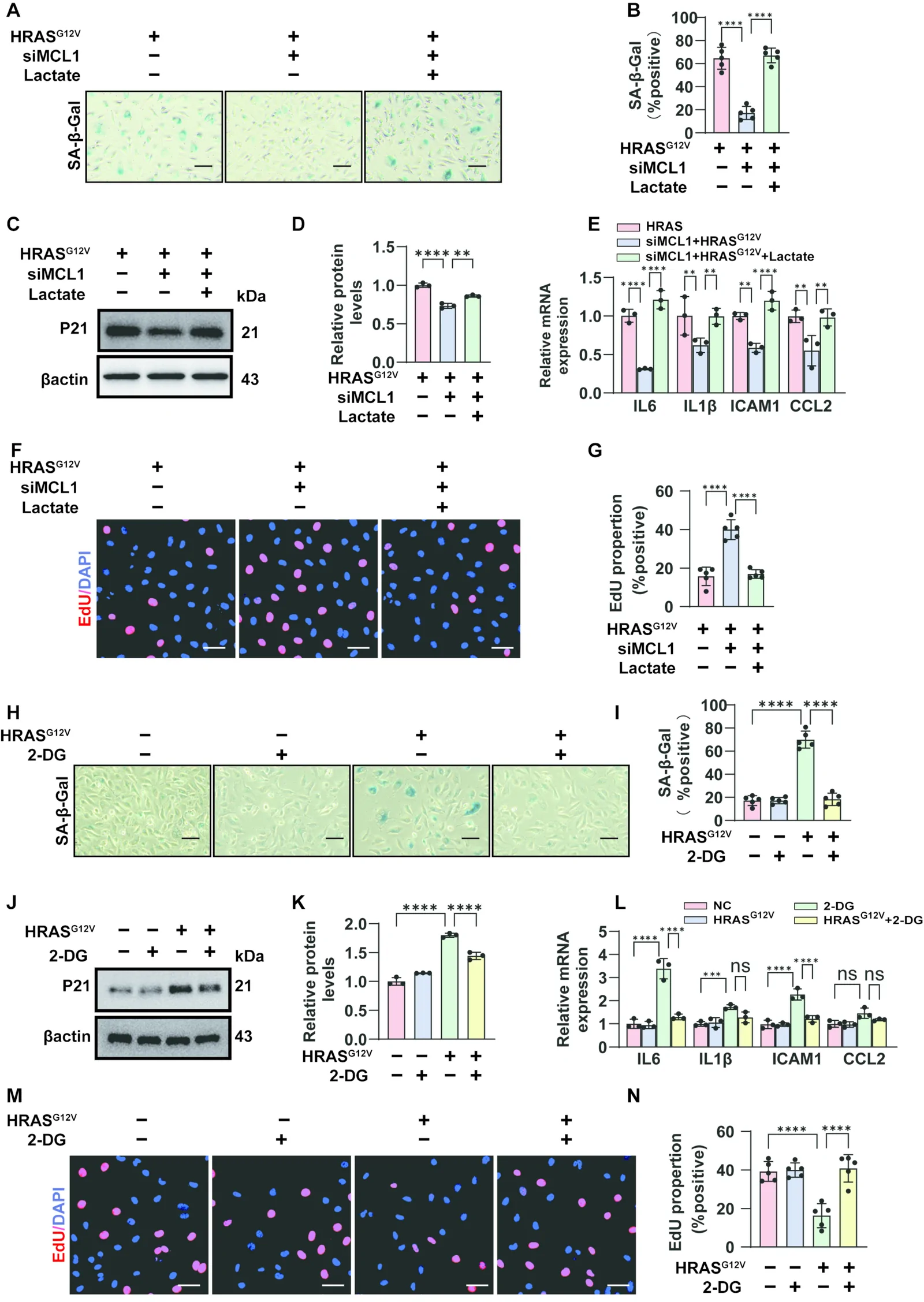
MCL1 promotes cellular senescence in HUVECs by mediating lactate accumulation. (**A**,**B**) Representative images (**A**) and quantification (**B**) of SA-β-gal staining in senescence-induced HUVECs with MCL1 knockdown (siMCL1), treated with or without exogenous sodium lactate (NaLa, 10 mM) for 24 h. The addition of NaLa abrogated the anti-senescent effect of siMCL1. (*n* = 5 independent biological replicates). Scale bar 100 μm. (**C**,**D**) Representative Western blot images (**C**) and densitometric quantification (**D**) of P21 protein expression in the specified groups. NaLa supplementation restored P21 expression despite MCL1 knockdown. (**E**) Relative mRNA expression of Senescence-Associated Secretory Phenotype (SASP) markers in senescence-induced HUVECs treated with siMCL1 and NaLa (10 mM, 24 h). (**F**,**G**) Representative images (**F**) and quantification (**G**) of EdU incorporation assays. NaLa treatment reduced the proliferative capacity rescued by siMCL1. (*n* = 5 independent biological replicates) (DAPI, blue; EdU, red). Scale bar 50 μm. (**H**,**I**) Representative images (**H**) and quantification (**I**) of SA-β-gal staining in senescence-induced HUVECs treated with the glycolysis inhibitor 2-Deoxy-D-glucose (2-DG, 2 mM) for 24 h. Inhibition of glycolysis decreased the proportion of senescent cells. (*n* = 5 independent biological replicates). Scale bar 100 μm. (**J**,**K**) Representative Western blot images (**J**) and densitometric quantification (**K**) of P21 protein expression in senescence-induced HUVECs following 2-DG treatment. (**L**) Relative mRNA expression levels of SASP markers in senescence-induced HUVECs treated with 2-DG. (**M**,**N**) Representative images (**M**) and quantification (**N**) of EdU incorporation assays demonstrating that 2-DG treatment restored cell proliferation. (*n* = 5 independent biological replicates) (DAPI, blue; EdU, red). Scale bar 50 μm. All bars represent mean ± SD. ns, no significance; ** *p* < 0.01; *** *p* < 0.001; **** *p* < 0.0001. (**B**,**D**,**E**,**G**) were analyzed by one-way ANOVA followed by Tukey’s post hoc test. (**I**,**K**,**L**,**N**) were analyzed by two-way ANOVA followed by Tukey’s post hoc test.

Given that MCL1 knockdown decreased lactate production in senescent HUVECs, we investigated whether lactate mediates the pro-senescent effect of MCL1. Supplementation with exogenous sodium lactate (NaLa; 10 mM, 24 h) reversed the effects of MCL1 knockdown in senescence-induced HUVECs. Specifically, NaLa treatment restored SA-β-gal positivity (Figure 5A,B), rescued P21 protein expression (Figure 5C,D), reactivated SASP gene transcription (Figure 5E), and abrogated the proliferative recovery induced by MCL1 knockdown (Figure 5F,G).

To further validate the causal role of glycolysis-derived lactate in endothelial senescence, we treated senescent HUVECs with the glycolysis inhibitor 2-deoxy-D-glucose (2-DG; 2 mM, 24 h). The 2-DG treatment significantly reduced the proportion of SA-β-gal-positive cells (Figure 5H,I), decreased P21 protein expression (Figure 5J,K), downregulated SASP mRNA levels (Figure 5L), and enhanced EdU incorporation in senescent HUVECs (Figure 5M,N).

To rule out potential non-specific or off-target effects of chemical inhibition and provide precise genetic evidence, we specifically targeted endogenous lactate synthesis by silencing LDHA (Figure S2A). Strikingly, LDHA knockdown in senescent HUVECs perfectly mirrored the anti-senescent effects of 2-DG. Depletion of LDHA substantially downregulated P21 protein accumulation (Figure S2B), diminished SA-β-gal activity (Figure S2C,D), restored cellular proliferative capacity (Figure S2E,F), and attenuated SASP transcription (Figure S2G).

Taken together, these data indicate that MCL1 promotes endothelial senescence through glycolytic lactate accumulation.

### 2.6. MCL1 1. Promotes HUVEC Senescence via H4K12 Lactylation

**Figure 6:**
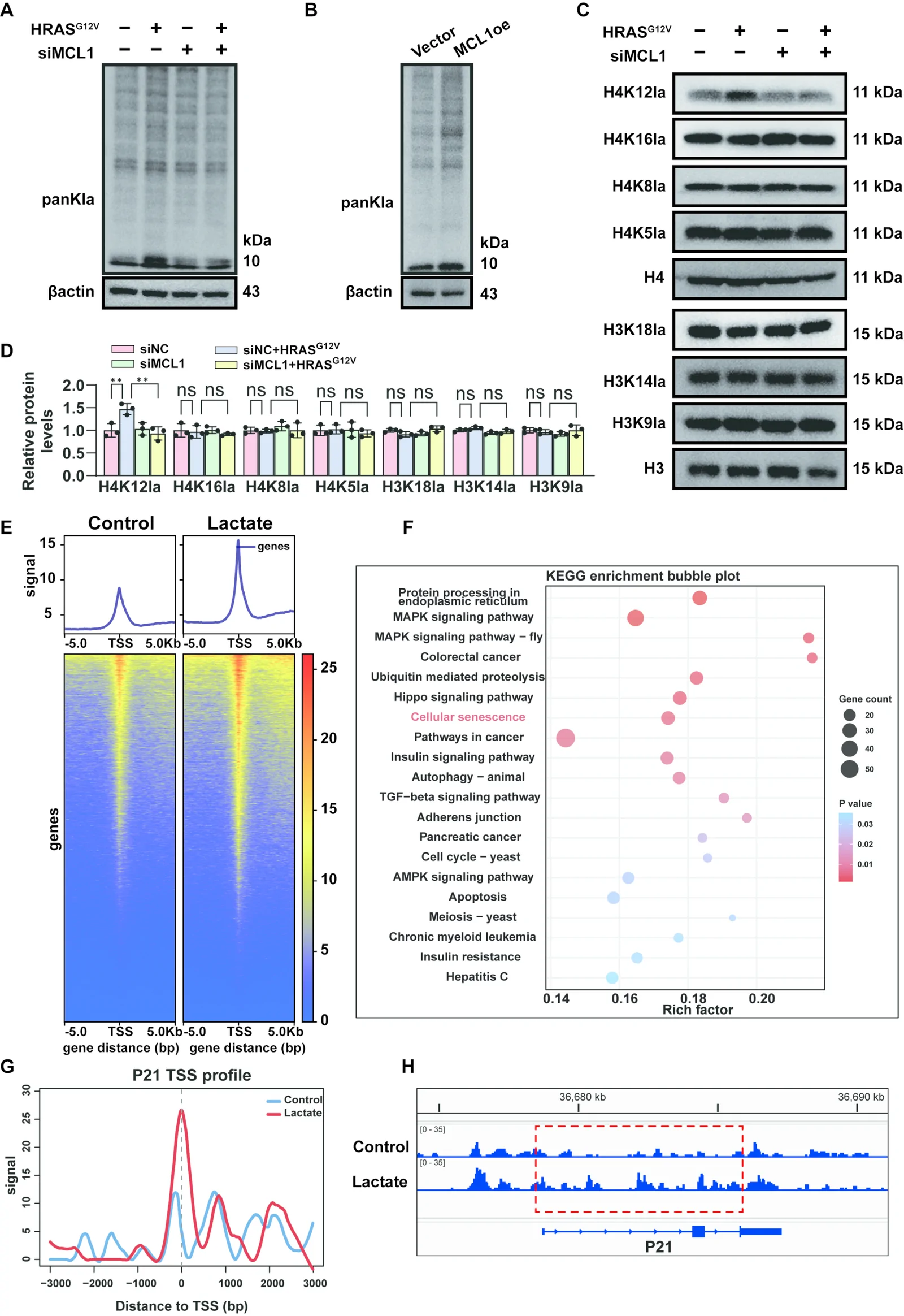
MCL1 promotes endothelial senescence through epigenetic regulation via histone H4K12 lactylation. (**A**,**B**) Pan-Kla is elevated in senescence-induced HUVECs and is reduced by genetic inhibition of MCL1 (siMCL1) (**A**). In contrast, overexpression of MCL1 elevates pan-lactylation levels in HUVECs (**B**). (**C**,**D**) Immunoblotting analysis of specific histone lactylation sites (including H4K12la, H4K16la, H4K8la, H4K5la, H3K18la, H3K14la, and H3K9la). H4K12la, H4K16la, H4K8la and H4K5la were normalized to total H4, and H3K18la, H3K14la and H3K9la were normalized to total H3. H4K12la levels were robustly upregulated in senescent HUVECs and subsequently decreased following MCL1 knockdown (H4K12la, siNC vs. siNC + HRAS^G12V^, *p* = 0.0033, siNC + HRAS^G12V^ vs. siMCL1 + HRAS^G12V^, *p* = 0.0015), whereas other specific lactylation sites exhibited no significant alterations. (**E**,**F**) CUT&Tag sequencing analysis comparing lactate-treated HUVECs with the control group (*n* = 2 independent biological replicates). (**E**) Heatmap illustrating the genome-wide enrichment of H4K12la signals across transcription start sites (TSS). NaLa (10 mM, 24 h) treatment markedly increased H4K12la binding globally. (**F**) KEGG pathway enrichment analysis revealing that genes with upregulated H4K12la binding in the lactate group were predominantly enriched in the “Cellular senescence” signaling pathway. (**G**,**H**) Visualization of H4K12la CUT&Tag sequencing tracks (peaks) at the promoter region of the CDKN1A gene (encoding P21). Exogenous lactate treatment substantially enhanced H4K12la enrichment at the CDKN1A promoter compared to the control group. All bars represent mean ± SD. ns, no significance; ** *p* < 0.01; (**D**) were analyzed by two-way ANOVA followed by Tukey’s post hoc test.

Lactate has recently been identified as a substrate for histone lactylation, a novel epigenetic modification that regulates gene expression. We therefore investigated whether MCL1-driven lactate accumulation promotes endothelial senescence through histone lactylation. Western blot analysis revealed that global protein lactylation (pan-Kla) levels were markedly elevated in senescent HUVECs, an increase that was abolished by MCL1 knockdown (Figure 6A). Conversely, MCL1 overexpression enhanced pan-Kla levels in HUVECs (Figure 6B).

To identify the specific lactylation sites regulated by MCL1, we examined several histone lactylation marks. Among the sites assessed, H4K12 lactylation (H4K12la) was significantly increased in senescent HUVECs and markedly reduced upon MCL1 knockdown, whereas H4K16la, H4K8la, H4K5la, H3K18la, H3K14la, and H3K9la levels remained unaltered (Figure 6C,D). These results identify H4K12la as a specific downstream target of MCL1-mediated lactate signaling.

To profile the genome-wide distribution of H4K12la, we performed CUT&Tag analysis in HUVECs treated with or without exogenous lactate. Heatmap analysis revealed a global increase in H4K12la enrichment at transcription start sites (TSSs) upon lactate treatment (Figure 6E). KEGG pathway enrichment analysis of genes with lactate-induced H4K12la deposition demonstrated significant enrichment in the cellular senescence pathway (Figure 6F). Notably, visualization of the *CDKN1A* (*P21*) locus revealed a markedly higher H4K12la peak at the *P21* promoter region in the lactate-treated group compared with the control group (Figure 6G,H), which is highly consistent with the transcriptional upregulation of P21 observed in our earlier experiments. Given our preceding data demonstrating that MCL1 drives glycolytic lactate production, these CUT&Tag results strongly support a mechanistic model in which MCL1 promotes endothelial senescence through a lactate-mediated H4K12la epigenetic regulation at the P21 promoter.

### 2.7. AAV-Mediated MCL1 Knockdown Reduces Endothelial H4K12la in Atherosclerotic Mice

**Figure 7:**
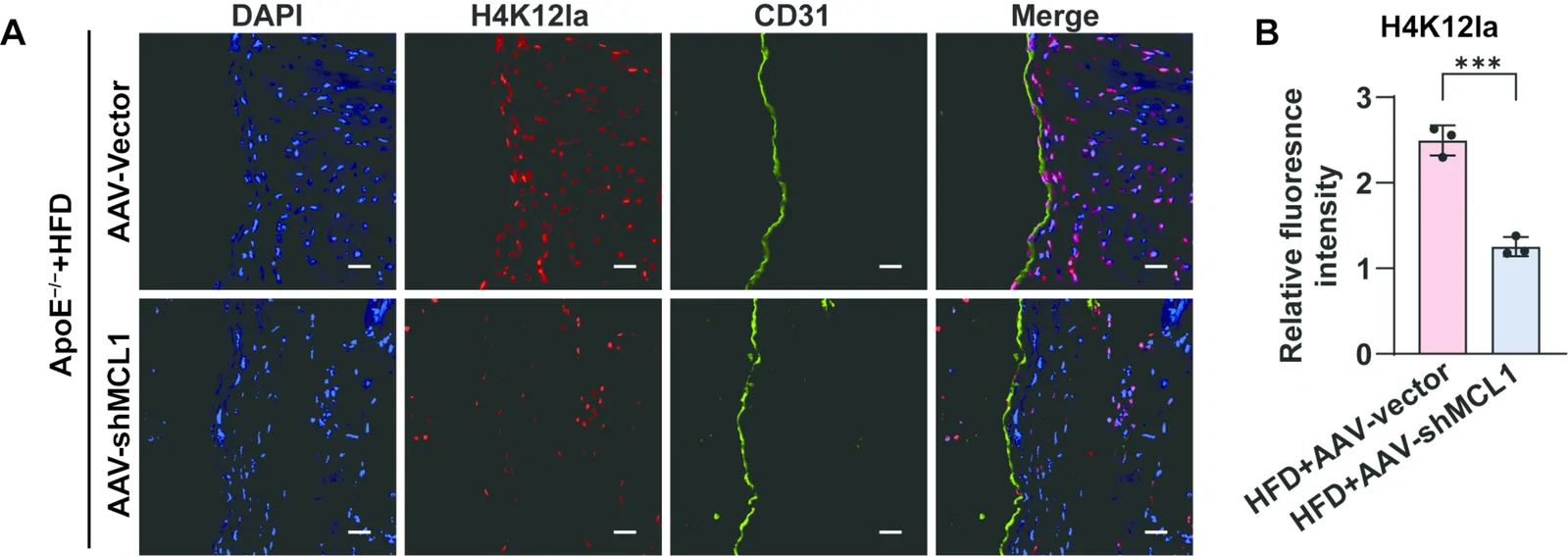
AAV-mediated knockdown of MCL1 decreases H4K12 lactylation in endothelial cells of atherosclerotic mice. (**A**,**B**) Representative immunofluorescence images (**A**) and quantification within CD31^+^ endothelial regions (**B**) demonstrating reduced H4K12 lactylation (H4K12la) levels in the vascular endothelial cells of atherosclerosis-induced ApoE^−/−^ mice following AAV-mediated MCL1 knockdown. (AAV-vector vs. AAV-shMCL1, *p* = 0.0005) (*n* = 3 independent biological replicates) (DAPI, blue; MCL1, green; CD31, red). Scale bar 100 μm. All bars represent mean ± SD. *** *p* < 0.001 (**B**) were analyzed by unpaired *t*-test.

To validate the regulatory relationship between MCL1 and H4K12la in vivo, we examined H4K12la levels in the vascular endothelium of atherosclerotic ApoE^−/−^ mice. Immunofluorescence staining revealed that systemic AAV-mediated MCL1 knockdown significantly decreased H4K12la levels specifically within the CD31-positive in vascular endothelial cells compared with AAV-vector mice (Figure 7A,B). These findings confirm that MCL1 modulates endothelial H4K12la deposition in vivo and provide a strong in vivo relevance for the metabolic–epigenetic cascade linking MCL1, histone lactylation, and endothelial senescence in atherosclerosis.

## 3. Discussion

Atherosclerosis is a chronic inflammatory disease closely associated with vascular aging [34]. Endothelial cell senescence has emerged as a central driver of endothelial dysfunction, plaque formation, and disease progression [6,35]. The present study identifies MCL1 as a previously unrecognized key regulator that links metabolic reprogramming, histone lactylation, and endothelial senescence in atherosclerosis.

Our data show that MCL1 expression is markedly elevated in both human and murine atherosclerotic lesions as well as in oncogene-induced senescent endothelial cells and oxidative stress-induced (TBHP) senescence models, confirming its relevance across distinct physiological and pathological senescence triggers. In vitro gain- and loss-of-function experiments indicate that MCL1 is both sufficient and necessary for establishing and maintaining the senescent phenotype. Given that MCL1 is a classic anti-apoptotic protein, a potential concern is that its depletion might simply induce non-specific cell death rather than true senescence reversal. However, our data showed that MCL1 knockdown restored EdU incorporation—a strict indicator of active DNA synthesis and viable cellular machinery—suggesting a functional restoration of the cell cycle rather than extensive cytotoxicity. Furthermore, a recent study demonstrated that selective MCL1 inhibition alone is insufficient to induce robust apoptosis in senescent endothelial cells, which typically require combined inhibition of multiple BCL-2 family members to undergo apoptosis [36]. When we used AAV-mediated knockdown of MCL1 in ApoE^−/−^ mice, plaque burden was substantially reduced, endothelial senescence was attenuated, and vascular inflammation decreased. These results suggest that MCL1 may play a causal role in disease progression in vivo.

Mechanistically, we found that MCL1 promoted a metabolic shift toward glycolysis in senescent endothelial cells, leading to increased lactate production. Specifically, our findings revealed that MCL1 physically interacts with and selectively maintains the protein expression of PFKP, a crucial rate-limiting enzyme in glycolysis. This lactate accumulation elevated H4K12la, a recently identified epigenetic modification. CUT&Tag analysis further demonstrated that H4K12la was markedly enriched at the CDKN1A (P21) promoter in senescent cells, which was strongly associated with enhanced P21 transcription. Pharmacological supplementation with sodium lactate and inhibition of glycolysis, as well as specific genetic silencing of endogenous LDHA, further confirmed that lactate serves as a critical metabolic signal linking MCL1 to P21-dependent senescence. These data suggest a potential metabolic–epigenetic link wherein MCL1-driven metabolic rewiring converges with epigenetic regulation to reinforce endothelial senescence via P21.

While our study primarily focused on endothelial cells, MCL1 is broadly expressed across multiple vascular cell types, and its regulation likely influences atherosclerosis progression through mechanisms extending beyond the endothelium. In our in vivo immunofluorescence analysis, MCL1 expression was also observed in non-endothelial cells within the plaque, likely representing vascular smooth muscle cells (VSMCs) and infiltrating macrophages (foam cells). Given that systemic delivery of AAV limits cell-type specificity, the marked reduction in atherosclerotic lesions observed in shMcl1-treated mice is likely a synergistic outcome involving these other vascular cell types. MCL1 is a well-known anti-apoptotic and pro-proliferative factor. Pathological VSMC proliferation and migration are primary drivers of plaque progression. Therefore, downregulation of MCL1 in VSMCs could potentially restrain their aberrant proliferation, contributing to the reduction in plaque volume. Similarly, MCL1 plays a critical role in macrophage survival. Its localized inhibition might modulate foam cell accumulation within the intimal space. However, it must be emphasized that MCL1 is a critical survival protein in multiple normal tissues. Systemic suppression of MCL1 may lead to undesirable generalized toxicities, highlighting the necessity for developing endothelial-targeted delivery systems (e.g., targeted nanoparticles) for any future translational applications. Although further studies utilizing endothelial-, VSMC-, and macrophage-specific conditional knockout models (e.g., Cdh5-Cre, Sm22-Cre, and Lyz2-Cre) are warranted to dissect these distinct cellular contributions, our current findings highlight systemic MCL1 modulation as a potent, multi-target atheroprotective strategy.

Several limitations should be noted. Although we observed increased MCL1 expression in human atherosclerotic specimens, the sample size was relatively small and corresponding clinical outcome data were unavailable. The AAV vectors used in the animal studies, while effective, do not confer strict endothelial cell specificity; therefore, off-target modulation of MCL1 in other vascular cell types cannot be excluded. In addition, although we utilized 2-DG and LDHA knockdown to validate the role of glycolysis, additional studies utilizing specific lactate-transporter inhibitors are warranted. Consequently, regarding the epigenetic mechanism, while our CUT&Tag profiling under lactate treatment showed an association between H4K12la and the CDKN1A promoter, we did not perform CUT&Tag directly following MCL1 manipulation, nor did we definitively prove causality using locus-specific ChIP-qPCR or functional H4K12la-specific mutants. Thus, the exact causal linearity of the MCL1–H4K12la–P21 connection requires further rigorous locus-specific validation. Moreover, we did not systematically evaluate classical apoptosis markers (e.g., Annexin V, cleaved caspase-3) or an expanded panel of senescence markers (e.g., P16, Lamin B1), which will be necessary to comprehensively distinguish anti-senescent effects from viability changes. Finally, while we identified an interaction between MCL1 and PFKP; whether MCL1 directly regulates PFKP enzymatic activity, protein stability, or subcellular localization remains to be fully elucidated.

In conclusion, our study demonstrates that MCL1 promotes endothelial senescence and atherosclerosis by enhancing lactate production and H4K12la deposition, ultimately associated with P21 activation. These findings not only expand our understanding of the interplay between metabolism and epigenetics in vascular aging but also suggest that targeting MCL1 may represent a novel mechanistic paradigm for mitigating endothelial senescence and atherosclerotic cardiovascular disease. Future studies employing more specific endothelial targeting tools and larger clinical cohorts will be required to fully validate this pathway and its translational potential.

## 4. Materials and Methods

### 4.1. Patient Specimens

This study was approved by the Ethics Committee of Zhongshan Hospital Qingpu Branch of Fudan University on 10 May 2022 (Approval No. 2022-38). All procedures conformed to the principles outlined in the Declaration of Helsinki. All participants were informed of the study protocol and provided written informed consent. Human carotid atherosclerotic plaques (*n* = 7) were obtained from patients with severe carotid stenosis who underwent carotid endarterectomy (CEA) at the Department of Vascular Surgery, Qingpu Branch of Zhongshan Hospital, between June 2024 and January 2025. Surgically resected tissues were immediately rinsed with ice-cold PBS to remove residual blood and superficial thrombi. Specimens were meticulously dissected to separate the central atherosclerotic lesion from the surrounding, visually normal, non-plaque intima. Samples were either fixed in 4% paraformaldehyde (PFA) or stored at −80 °C for subsequent analysis. In addition, corresponding baseline patient profiles, comprising age, sex, and pre-existing comorbidities, were systematically collected (Table 1). Four samples collected earlier had undergone prolonged storage conditions, which led to severe antigen masking and autofluorescence, rendering them unsuitable for high-quality immunofluorescence imaging. Conversely, three subsequently and freshly collected specimens optimally preserved tissue architecture and antigenicity. These three samples were utilized for immunofluorescence staining. Western blot analyses were performed using all seven patient samples.

### 4.2. Animal Experiments

For establishment of the atherosclerosis model, male ApoE^−/−^ mice on a C57BL/6 background (8 weeks of age) were purchased from Hangzhou Ziyuan Experimental Animal Technology Co., Ltd (Hangzhou, China). The mice were housed under specific pathogen-free (SPF) conditions with a 12 h light/dark cycle. Animals were randomly assigned to four groups (*n* = 6 per group): Control, HFD, HFD + AAV-vector, and HFD + AAV-shMCL1. All animals had ad libitum access to water throughout the treatment period. Mice in the Control group were fed a standard chow diet, whereas mice in the HFD, HFD + AAV-vector, and HFD + AAV-shMCL1 groups received a high-fat diet (HFD; D12109C, Research Diets) for 12 weeks. To investigate the role of MCL1 in endothelial senescence, scAAV2/9-U6-shRNA (mMCL1)-CMV-ZsGreen (5′-AAACGAAGACGATGTGAAATC-3′) and scAAV2/9-U6-shRNA (NC)-CMV-ZsGreen (5′-CCTAAGGTTAAGTCGCCCTCG-3′), obtained from YiXueSheng Biosciences Inc. (Shanghai, China), were administered via tail vein injection during the final 8 weeks of the 12-week HFD feeding period. Upon completion of the protocol, all mice were euthanized by CO_2_ inhalation, and aortic tissues were collected from the 6 mice in each group. Tissues from 3 randomly selected mice were subjected to immunofluorescence staining, and those from the other 3 mice were used for Western blot analysis.

### 4.3. Reagents and Antibodies

Sodium lactate (NaLa; HY-B2227B) and 2-deoxy-D-glucose (2-DG; HY-13966) were purchased from MedChemExpress (MCE, Shanghai, China). Puromycin (S7417), polybrene (E1299), and Protein A/G magnetic beads (B23202) were obtained from Selleck (Houston, TX, USA). Oil Red O (234117) was purchased from Sigma-Aldrich (St. Louis, MO, USA). Diaminobenzidine (DAB) substrate kits (G1212-200T), PMSF (G2008-1 mL) and phosphatase inhibitors (G2007-1 mL) were obtained from Servicebio (Wuhan, China). TRIzol reagent (15596018) was purchased from Thermo Fisher Scientific (Waltham, MA, USA). PrimeScript™ RT Master Mix (RR036B) was obtained from Takara (Kusatsu, Japan). A 2× Color SYBR Green qPCR Master Mix (A0012-R2) was purchased from EZBioscience (Suzhou, China). Western blot and IP lysis buffer (P0013J), Lipo8000™ Transfection Reagent (C0533-7.5 mL), and DAPI (C1002) were obtained from Beyotime (Shanghai, China). The EdU-555 Cell Proliferation Kit (CX003) was purchased from Epizyme (Shanghai, China). The XF Cell Mito Stress Test Kit (103015-100) and XF Glycolysis Rate Assay Kit (103344-100) were obtained from Agilent Technologies (Santa Clara, CA, USA). The Hyperactive Universal CUT&Tag Assay Kit (TD904) for Illumina Pro was purchased from Vazyme (Nanjing, China). The CheKine™ Micro Lactate Assay Kit (KTB1100) was obtained from Abbkine (Wuhan, China).

Antibodies against MCL1 (1:1000, 16225-1-AP), CD31 (1:1000, 66065-2-Ig), and P21 (1:1000, 10355-1-AP and 67362-1-Ig) were purchased from Proteintech (Wuhan, China). Antibodies against H3K9la (1:1000, PTM-1419RM), H3K14la (1:1000, PTM-1414RM), H3K18la (1:1000, PTM-1427RM), H4K12la (1:1000, PTM-1411RM), H3 (1:3000, PTM-1001RM), and H4 (1:3000, PTM-1015RM) were obtained from PTM BIO (China). The β-actin antibody (1:1000, ZB15001-HRP-100) was purchased from Servicebio (Wuhan, China).

### 4.4. Cell Culture

Human umbilical vein endothelial cells (HUVECs) were purchased from Shanghai Zhongqiao Xinzhou Biotechnology Co., Ltd. (Shanghai, China). Cells were cultured in Dulbecco’s Modified Eagle Medium (DMEM) supplemented with 10% fetal bovine serum (FBS) and 1% penicillin–streptomycin (100 U/mL penicillin and 100 μg/mL streptomycin). Cells were maintained at 37 °C in a humidified incubator containing 5% CO_2_ and were passaged using trypsin–EDTA. The cell line was authenticated by short tandem repeat (STR) profiling and confirmed to be mycoplasma-free prior to experimentation.

### 4.5. Plasmids and Transfection

Transfection was performed by complexing 2.5 μg of plasmid DNA with 4 μL of Lipofectamine 8000 in 125 μL of Opti-MEM. Following gentle mixing, the resulting liposome–DNA complexes were added dropwise and evenly into the 6-well culture plates. Cells were then cultured for an additional 24–48 h prior to lysis or harvesting for downstream experimental analyses. The MCL1-expression plasmid (pCDH-CMV-MCL1-3×Flag-EF1-CopGFP-T2A-Puro) and siMCL1 (5′-AAACGAAGACGAUGUGAAA-3′; 3′-UUUCACAUCGUCUUCGUUU-5′) were purchased from YiXueSheng Biosciences Inc. (Shanghai, China).

### 4.6. Cellular Senescence Model

HEK-293T cells were pre-cultured for 24 h. We then transfected them with 20 μg of the HRAS^G12V^ vector (WZ Bioscience Inc. Shandong, China). After a 15 h incubation at 37 °C, the first virus-containing supernatant was harvested and clarified through a 0.45 μm filter (Millipore, Billerica, MA, USA). A second viral harvest was performed on the same schedule. For target cell transduction, HUVECs were exposed to an optimized mixture of the first viral filtrate and DMEM for 12 h at 37 °C. We immediately repeated the infection cycle with the second viral harvest. Stable transformants were established through puromycin selection (1 μg/mL) for 72 h.

### 4.7. Seahorse Assay

Cellular metabolic flux was evaluated with the Seahorse XF Cell Mito Stress Test Kit and the Glycolysis Rate Assay Kit. We seeded HUVECs in Seahorse XF microplates at 8000 cells/well and let them adhere overnight. On the day of the assay, cells were maintained in Seahorse XF Base Medium supplemented with 10 mM glucose, 2 mM glutamine, and 1 mM sodium pyruvate. In parallel, sensor cartridges were pre-hydrated with XF Calibrant at 37 °C in a CO_2_-free environment overnight. The designated pharmacological modulators were loaded into the corresponding ports of each cartridge. To assess the glycolytic rate, rotenone/antimycin A (Rot/AA) and 2-deoxy-D-glucose (2-DG) were injected to achieve final working concentrations of 0.5 μM and 50 mM, respectively. For the mitochondrial stress assay, cells were sequentially treated with 2.5 μM oligomycin, 2 μM FCCP, and 0.5 μM Rot/AA. Following the assays, cell nuclei were stained with DAPI and cell numbers in each well were quantified using an Agilent BioTek Cytation 1 Cell Imaging Multi-Mode Reader (Agilent Technologies, Santa Clara, CA, USA), Raw metabolic rates were normalized and expressed per 10,000 cells. A total of 3 technical replicate wells were analyzed per condition in each run, and the average was taken as one biological sample. The entire experiment was independently repeated 5 times. Statistical analyses were performed based on these 5 independent biological replicates. Data were analyzed and calculated using the Agilent Seahorse Wave software(version 2.6.4.24).

### 4.8. Western Blot

We extracted total proteins using a 1% SDS lysis buffer supplemented with PMSF and phosphatase inhibitors. Equal amounts of protein were resolved on 10% or 12% SDS-PAGE gels and transferred to PVDF membranes. Membranes were blocked in 5% non-fat dry milk for 60 min at room temperature to prevent non-specific binding, then incubated with primary antibodies overnight at 4 °C. We washed the membranes three times in TBST (5 min each) before incubating them with the corresponding secondary antibodies for 1 h at room temperature. A final series of TBST washes preceded signal detection on a chemiluminescence imaging system, and densitometric analysis was carried out in ImageJ (version 1.54p).

### 4.9. Real-Time Quantitative Polymerase Chain Reaction (RT-qPCR)

Total RNA was extracted from cultured cells using TRIzol reagent (Invitrogen, Thermo Fisher Scientific, Inc., Waltham, MA, USA). Reverse transcription into cDNA was performed with PrimeScript RT Master Mix (Cat. No. RR036A; Takara Bio, Inc., Kusatsu, Japan). For quantitative real-time PCR, reaction mixtures were prepared with SYBR-Green PCR Master Mix (EZBioscience, Suzhou, China) and analyzed on an ABI 7500 Real-Time PCR System. Relative gene expression was calculated using the 2^−ΔΔCT^ method, with β-actin serving as the endogenous control. The primers used in this study were shown in Table 2. All assays were performed in triplicate, and the mean expression value of the control group was normalized to 1.0.

### 4.10. Immunofluorescence

For immunofluorescence analysis, both cultured HUVECs and aortic root cryosections were fixed in 4% paraformaldehyde for 10 min and subsequently permeabilized with 0.3% Triton X-100 for 15 min at room temperature. To reduce non-specific antibody binding, we used different blocking conditions. Cell samples were incubated in 5% sheep serum for 1.5 h, while tissue sections were blocked in 10% BSA for 1 h at room temperature. After blocking, we incubated all samples with the designated primary antibodies overnight at 4 °C. We then washed the specimens three times for 5 min each in PBST before adding the corresponding fluorophore-conjugated secondary antibodies for 1.5 h in the dark at room temperature. Nuclei were counterstained with DAPI for 15 min. After a final series of PBST washes, fluorescent signals were acquired using an Olympus laser-scanning confocal microscope.

### 4.11. SA-β-Gal Staining

To assess cellular senescence, HUVECs were seeded at a sub-confluent density to preclude density-induced false-positive staining. Cells were fixed for 15 min at room temperature, thoroughly rinsed with PBS, and subsequently incubated with freshly prepared SA-β-gal staining solution. Incubation was carried out overnight in the dark at 37 °C in a CO_2_-free environment. Representative bright-field images were captured, and the proportion of positively stained cells was quantified using ImageJ software. For long-term preservation, specimens were overlaid with 70% glycerol and stored at 4 °C.

### 4.12. 5-Ethynyl-2′-Deoxyuridine (EdU) Staining

EdU incorporation was assessed using the EdU-555 Cell Proliferation Kit (Epizyme, Shanghai, China). Cells were seeded onto 35 mm confocal dishes (NEST, 801001) and pulse-labeled with the EdU working solution at 37 °C for 2 h. After labeling, cells were fixed, rinsed, and permeabilized in accordance with the manufacturer’s instructions, followed by click-chemistry detection using a reaction mixture containing CuSO_4_, 555 azide, Click Additive Solution, and Click Reaction Buffer. Nuclei were counterstained with DAPI for 15 min, and images were acquired using an Olympus laser-scanning confocal microscope.

### 4.13. CUT&Tag Assay

CUT&Tag was performed with the Hyperactive Universal CUT&Tag Assay Kit(Vazyme, Nanjing, China) for Illumina Pro in accordance with the manufacturer’s instructions. HUVECs were bound to concanavalin A-coated magnetic beads, permeabilized, and incubated with an anti-H4K12la primary antibody. A Protein A/G–Tn5 Pro transposome was subsequently applied to catalyze on-target tagmentation. The resulting DNA fragments were PCR-amplified to generate barcoded, Illumina-compatible libraries, which were pooled and sequenced on an Illumina NovaSeq X platform (Illumina, San Diego, USA) with 150 bp paired-end reads (PE150). The sequencing depth was set at 6 G per sample for the CUT&Tag libraries. The sequencing data generated in this study have been deposited in the NCBI Sequence Read Archive (SRA) under accession number PRJNA1481600.

### 4.14. Oil Red O Staining

Atherosclerotic plaque burden was assessed by Oil Red O (ORO) staining. Briefly, isolated whole aortas were longitudinally dissected, fixed in 4% paraformaldehyde, and stained with ORO working solution to visualize en face lipid accumulation. Similarly, OCT-embedded aortic roots were cryosectioned (8 μm) and subjected to ORO staining. Background dye was removed with 60% isopropanol. Plaque areas were subsequently imaged and quantified using ImageJ software.

### 4.15. Lactate Assay

Intracellular lactate content was determined using the CheKine™ Micro Lactate Assay Kit (Abbkine, Wuhan, China). Briefly, cells were harvested, rinsed twice with ice-cold PBS, and lysed in the extraction buffer provided with the kit. Following centrifugation at 12,000× *g* for 10 min at 4 °C, the supernatant was collected and incubated with the assay working solution at 37 °C for 30 min. Absorbance was measured at 450 nm using a microplate reader, and lactate concentrations were calculated based on a standard curve generated in parallel.

### 4.16. Statistical Analysis

All statistical analyses were performed using GraphPad Prism software (version 10.0). Data are presented as the mean ± standard deviation (SD). Comparisons between two groups were performed using an unpaired Student’s *t*-test, and comparisons among multiple groups were performed using one-way or two-way analysis of variance (ANOVA) followed by Tukey’s post hoc test. A *p*-value < 0.05 was considered statistically significant.

## Figures and Tables

**Table 1:** Baseline data for patients used in this study.

	AS Patients						
Age	70	62	63	68	73	61	67
Sex	Men	Men	Female	Female	Men	Men	Men
Total cholesterol (mmol/L)	4.25	3.41	2.74	3.82	4.49	3.68	4.06
LDL cholesterol (mmol/L)	2.32	1.84	1.91	2.19	2.25	1.77	2.04
Triglycerides (mmol/L)	1.17	1.47	0.85	1.06	1.52	1.87	1.92
Hypertension	Yes	Yes	No	No	Yes	Yes	No
Diabetes Mellitus	No	Yes	Yes	No	Yes	No	Yes
Medication Usage							
Statin	Yes	Yes	Yes	Yes	Yes	Yes	Yes
Aspirin	Yes	Yes	Yes	Yes	Yes	Yes	Yes

**Table 2:** RT-qPCR primers used in this study.

Gene Name	Forward Sequence	Reverse Sequence
Human		
MCL1	CCAAGAAAGCTGCATCGAACCAT	CAGCACATTCCTGATGCCACCT
β-actin	GCGAGAAGATGACCCAGATC	CCAGTGGTACGGCCAGAGG
IL-6	AGACAGCCACTCACCTCTTCAG	TTCTGCCAGTGCCTCTTTGCTG
IL-1β	CCACAGACCTTCCAGGAGAATG	GTGCAGTTCAGTGATCGTACAGG
ICAM-1	AGCGGCTGACGTGTGCAGTAAT	TCTGAGACCTCTGGCTTCGTCA
CCL2	AGAATCACCAGCAGCAAGTGTCC	TCCTGAACCCACTTCTGCTTGG

## Data Availability

Senescence-associated genes obtained from CellAge database (https://genomics.senescence.info/cells/ (accessed on 10 December 2024)), mitochondrial proteins obtained from MitoMiner database (http://mitominer.mrc-mbu.cam.ac.uk/ (accessed on 10 December 2024)). GSE283095 obtained from GEO database (https://www.ncbi.nlm.nih.gov/geo/ (accessed on 10 December 2024)). The CUT & Tag sequencing data generated in this study have been deposited in the NCBI Sequence Read Archive (SRA) (https://trace.ncbi.nlm.nih.gov/Traces/index.html (accessed on 30 July 2026)) under accession number PRJNA1481600.

## Supplementary Materials

The following supporting information can be downloaded at: https://www.mdpi.com/article/10.3390/ijms27167392/s1.
