# Supplementary material for: MCL1 Promotes Endothelial Senescence and Atherosclerosis via Glycolytic Reprogramming-Induced H4K12 Lactylation
Source: Int J Mol Sci. 2026 Aug 18;27(16):7392. doi: 10.3390/ijms27167392 (PMC13513169; doi:10.3390/ijms27167392)
Supplement: Supplementary file 1 [file ijms-27-07392-s001.zip › ijms-4466508-supplementary.pdf]

Supplement. Materials and Methods

TBHP-induced senescence in HUVECs

Tert-butyl hydroperoxide (TBHP) (B106035) was purchased from Aladdine Inc(China). HUVECs at passages 3-5 were seeded into 6-well plates one day before treatment to achieve 60–70% confluence at the time of induction. Cellular senescence was induced by exposure to TBHP 200 μM for 2 h. After treatment, the medium was replaced with fresh complete culture medium without TBHP, and cells were further cultured for 48 h before subsequent experiments.

Reagents and antibodies

LDHA (1:1000, 3582T), HK1 (1:1000, 2024T), HK2(1:1000, 2867T), PDH (1:1000, 3205T), PFKP (1:1000, 8164T), and PKM2 (1:1000, 4053T) were purchased from Cell Signaling Technology (USA). Oxidized low-density lipoprotein (oxLDL) (YB-002) was purchased from Yiyuan Biotechnologies (China).

Immunoprecipitation (Co-IP)

For the endogenous Co-IP analysis, 20 μL of Protein A/G magnetic beads were pre-washed with 1× TBS and split into two equal aliquots. These aliquots were respectively conjugated with 1.5 μg of specific anti-MCL1 antibody or non-immune control IgG. Cellular protein extracts were then added to the bead-antibody complexes and rotated overnight at 4°C to form immunoprecipitates. The targeted protein complexes were magnetically isolated, washed five times with 1× TBS to eliminate non-specific binding, and subsequently denatured at 95°C for 5 min in 50 μL of 1× loading buffer. The specific protein interactions were ultimately visualized using standard immunoblotting procedures.

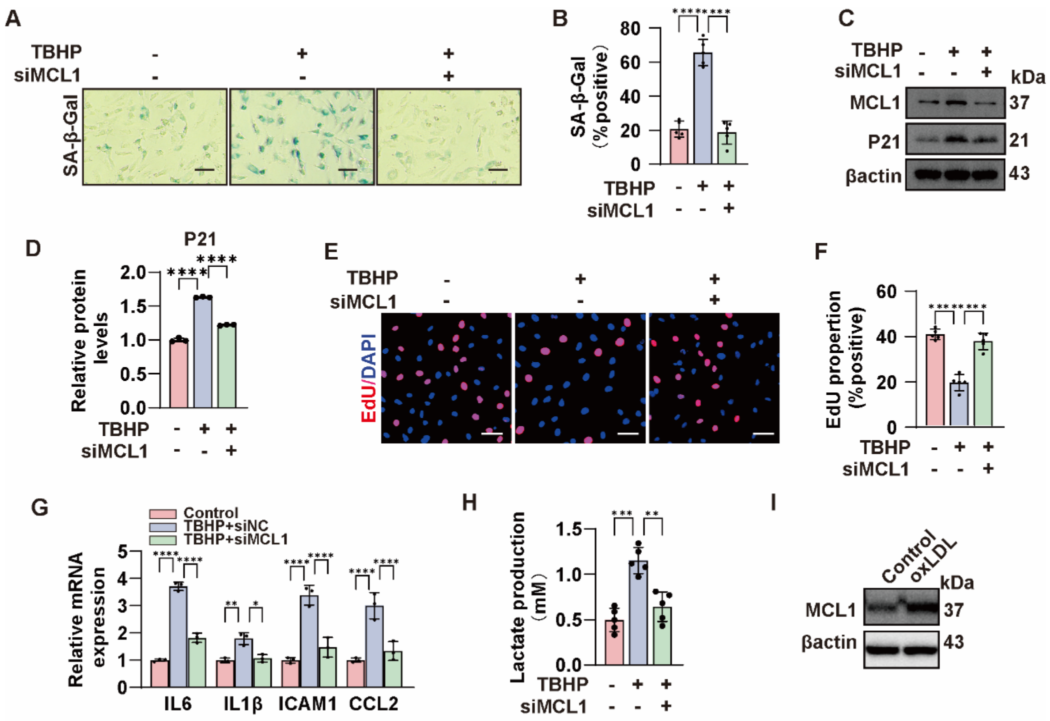

Figure S1. MCL1 promotes oxidative stress–induced endothelial senescence and glycolytic remodeling in HUVECs.

(A, B) Representative SA-β-gal staining images and quantification showing that tert-butyl hydroperoxide (TBHP) treatment (200 μM for 2 h followed by culture in fresh complete medium for 3 days) induced senescence in HUVECs, as evidenced by an increased percentage of SA-β-gal-positive cells. MCL1 knockdown reduced the proportion of SA-β-gal-positive cells. (n=5 independent biological replicates) .Scale bars 100 μm.

(C, D) Western blot analysis and quantification showing increased P21 and MCL1 protein expression in TBHP-induced senescent HUVECs, which was attenuated by MCL1 knockdown.

(E, F) Representative EdU staining images and quantification showing reduced proliferative capacity in TBHP-induced senescent HUVECs, whereas MCL1 knockdown increased the percentage of EdU-positive cells. (n=5 independent biological replicates) (DAPI, blue; EdU, red). Scale bar 50μm.

(G) qRT-PCR analysis showing increased transcriptional levels of SASP-associated genes (IL6, IL1β, ICAM1, and CCL2) following TBHP treatment, which were reduced after MCL1 knockdown.

(H) Lactate production assay showing increased lactate generation in TBHP-induced senescent HUVECs, which was decreased by MCL1 knockdown. (n= 5 independent biological replicates)

(I) Western blot analysis showing increased MCL1 protein expression in HUVECs treated with oxLDL (50 μg/L) for 48 h.

All bars represent Mean ± SD. ns, no significance; *P < 0.05; **P < 0.01; ***P < 0.001; ****P < 0.0001. (B),(D),(F) and (G) were analyzed by one-way ANOVA followed by Tukey's post hoc test.

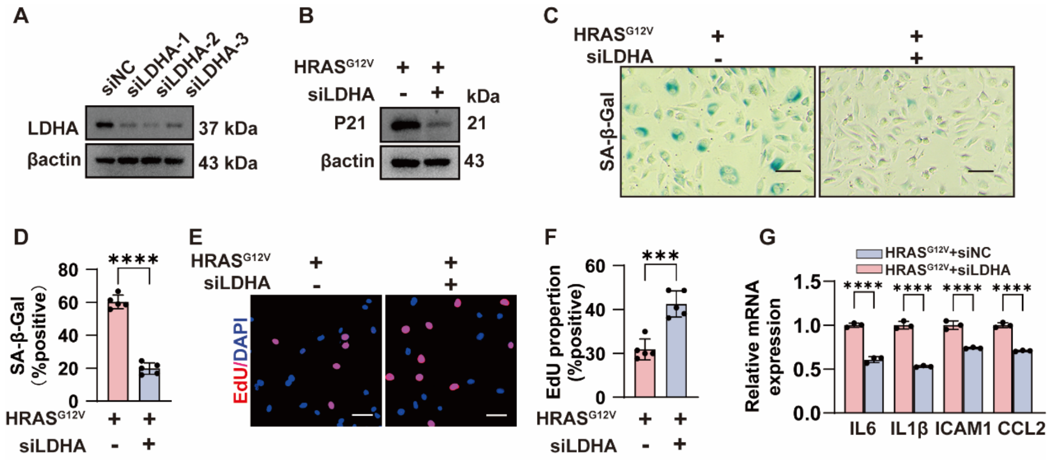

Figure S2. LDHA knockdown attenuates $HRAS^{G12V}$-induced endothelial senescence in HUVECs.

(A) Western blot analysis confirming the efficiency of LDHA knockdown by siRNA.

(B) Western blot analysis showing reduced p21 protein expression following LDHA knockdown in $HRAS^{G12V}$-induced senescent HUVECs.

(C, D) Representative SA-β-gal staining images and quantification demonstrating decreased proportions of SA-β-gal-positive cells after LDHA knockdown in $HRAS^{G12V}$-induced senescent HUVECs. (n=5 independent biological replicates) .Scale bars 100 μm.

(E, F) Representative EdU staining images and quantification showing increased proportions of EdU-positive cells following LDHA knockdown in $HRAS^{G12V}$-induced senescent HUVECs. (n=5 independent biological replicates) (DAPI, blue; EdU, red). Scale bar 50μm.

(G) qRT-PCR analysis showing reduced transcriptional levels of senescence-associated secretory phenotype (SASP) factors after LDHA knockdown in $HRAS^{G12V}$-induced senescent HUVECs.

All bars represent Mean ± SD. ns, no significance; $*P < 0.05$; $**P < 0.01$; $***P < 0.001$; $****P < 0.0001$. (D),(F) and (G) were analyzed by unpaired t-test.

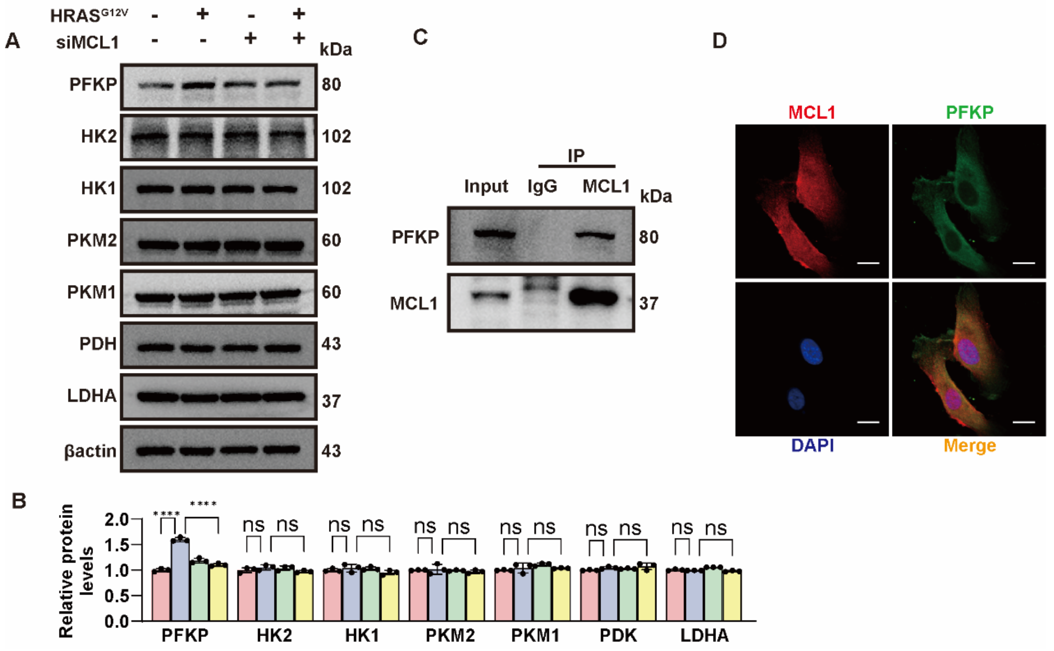

Figure S3. MCL1 interacts with PFKP and regulates its expression in senescent HUVECs.

(A,B) Representative Western blot (A) and quantitative analysis (B) demonstrating that MCL1 knockdown decreased PFKP protein expression in senescent HUVECs, while the expression levels of other glycolytic enzymes, including HK2, HK1, PKM2, PKM1, PDH, and LDHA, were not significantly altered.

(C) Co-immunoprecipitation (Co-IP) assay revealing a protein–protein interaction between MCL1 and PFKP in HUVECs.

(D)Representative immunofluorescence images showing co-localization of MCL1 (red) and PFKP (green) in HUVECs. Nuclei were stained with DAPI (blue). Scale bar 20μm.

All bars represent Mean ± SD. ns, no significance; $*P < 0.05$; $**P < 0.01$; $***P < 0.001$; $****P < 0.0001$. (B) were analyzed by two-way ANOVA followed by Tukey's post hoc test.
